# Co-Creating Snacks: A Cross-Cultural Study with Mediterranean Children Within the DELICIOUS Project

**DOI:** 10.3390/foods14020159

**Published:** 2025-01-07

**Authors:** Elena Romeo-Arroyo, María Mora, Olatz Urkiaga, Nahuel Pazos, Noha El-Gyar, Raquel Gaspar, Sara Pistolese, Angelique Beaino, Giuseppe Grosso, Pablo Busó, Juancho Pons, Laura Vázquez-Araújo

**Affiliations:** 1 BCC Innovation, Technology Center in Gastronomy, Basque Culinary Center, 20009 Donostia-San Sebastián, Spain; 2 Basque Culinary Center, Faculty of Gastronomic Sciences, Mondragon Unibersitatea, 20009 Donostia-San Sebastián, Spain; 3 Department of Pediatric, Faculty of Medicine, Assiut University, Assiut 71515, Egypt; 4 Provincia Portuguesa da Congregacao dos Irmaos Maristas, Estr. De Benfica 372, 1500-133 Lisbon, Portugal; 5 Provincia d’Italia dei Fratelli Maristi delle Scuole, Via Fratelli Maristi 2, 80014 Giugliano in Campania, Italy; 6 Freres Maristes au Liban, Dik el Mehdi, Beyrouth 70540, Lebanon; 7 Department of Biomedical and Biotechnological Sciences, University of Catania, 95123 Catania, Italy; 8 Center for Human Nutrition and Mediterranean Foods (NUTREA), University of Catania, 95123 Catania, Italy; 9 Technological Institute for Children’s Products & Leisure AIJU, 03440 Alicante, Spain; 10 Editorial Luis Vives (EDELVIVES), Carretera de Madrid, 50012 Zaragoza, Spain

**Keywords:** co-design, consumer study, sensory properties, emojis, acceptance

## Abstract

Mediterranean diet adherence has been decreasing during the last few decades, and non-appropriate snacking habits have also been identified among Mediterranean children and adolescents. To co-create new snacks and to explore children’s interests and preferences, a multi-method approach was used in the present study, including some qualitative and quantitative research phases. Conducted in collaboration with schools in Lebanon, Egypt, Portugal, Italy, and Spain, different snack prototypes were designed and tested in a Mediterranean cross-cultural context. The results showed significant differences among countries in snacking preferences and general food-related attitudes. Italian children exhibited higher levels of neophobia, resulting in lower acceptance of all proposed snacks. Some sensory and contextual insights were collected, such as Egyptian children favoring sweet and crunchy textures and “At school”, “With my friends”, and “As a morning/afternoon snack” being identified as linked to snack acceptance in some countries. The present study underscores the value of co-creation processes involving children to address non-recommended dietary patterns, highlighting the critical role of sensory properties, cultural differences, and contextual factors in designing healthy snacks that meet the Mediterranean diet’s principles but are highly appreciated by the young segment of the population.

## 1. Introduction

The Mediterranean diet (MD) has been suggested as an example of a healthy diet for decades and has therefore been promoted due to its cardioprotective and beneficial effects [1]. Because food choices have been reported to be shaped early in life [2], several nutritional programs have been designed and launched to investigate and increase MD adherence among children [3,4,5,6]. In general, high levels of physical activity and meal frequency, low screen time, and low scores on external eating behavior have been associated with higher adherence to the Mediterranean diet [4]. Alongside identifying deviations from the MD in general dietary patterns, some of the key findings from these studies have shown that participants’ snacking behaviors often diverge from the recommended MD guidelines, with children and adolescents often consuming fruits and vegetables but also consuming sugared beverages and different kinds of processed snacks (e.g., candies and salty snacks) [7,8].

Adherence to an unhealthy snacking pattern has been associated with a greater incidence of metabolic syndrome [9], suggesting that the quality of snacks, rather than simply their frequency, could play a crucial role in disease risk. Some hypotheses have been proposed to explain the shift in current snacking habits among children. Fernández San Juan [7] reported that Spanish children’s growing independence was contributing to a shift away from the traditional MD and towards unhealthy snacking habits, with them choosing processed foods with lower nutritional value. In addition, the external eating profile has been positively associated with the frequency of consumption of snack foods among children [10], alongside its aforementioned negative correlation with MD adherence [4]. External eaters are those who eat mainly driven by external stimuli, such as the mere presence of the food or its smell [11]. Therefore, promoting healthy snacking is crucial for reintroducing Mediterranean diet principles to young people, and increasing nutritious snack options could be a strategy through which to encourage external eaters to make healthier choices.

The concept of co-creation has gained traction in the field of food design, with researchers recognizing the value of actively engaging with consumers to gather their opinions, expectations, and beliefs throughout different phases (e.g., idea generation) of a new product’s development process [12,13]. Galler et al. [14,15] explored the feasibility of including preadolescents in co-creating healthy snacks, proposing a series of activities in which the young participants could propose new product ideas while researchers collected not only these ideas but additional insights linked to the psychosocial aspects of snacking. Alongside this approach to food design, codesign and co-creation activities with children have been proposed for the development of new games [16], outdoor play activities [17], and even health report cards [18]. Jenkins et al. [19] proposed co-design activities with adolescents to create new recipes from leftovers. These authors reported that adolescents not only enjoyed the intervention process but also developed a deeper understanding of the issue of food waste and its environmental implications, highlighting the potential of the research process as a learning-by-doing activity.

The aim of the present study was to deploy a co-creation activity in which children from different Mediterranean countries could express their opinions and ideas about different kinds of snacks in order to develop alternatives to the low-nutritional-quality ones they were currently consuming. The present research focused on developing new snacks, building upon findings from a previous study within the same European Project (DELICIOUS) [20]. The earlier study investigated the dietary habits of Mediterranean children, including their food consumption frequency, and identified key areas where their diets deviated from the principles of the Mediterranean diet. The results from the descriptive analysis on Mediterranean diet adherence of the children involved in the project indicated that only a minority of participants fulfilled the positive recommendation of “daily consumption of pasta, cereals, and nuts” from the KIDMED questionnaire and also suggested inadequate consumption of fruits and vegetables, as well as sweets and candies [21]. Therefore, the aim was to develop products in which some of these ingredients were included (e.g., nuts and cereals), trying to promote heathier snacking among Mediterranean children. In addition, the results of the aforementioned study indicated that the products most consumed by children who snacked were fruit (26%), and commercial biscuits and pastries (21%). Because fruits are perfect snacks that should not be replaced, the “commercial biscuits and pastries” category was the main target for improvement, with the aim of providing attractive substitutes for the products available on the market. As well as providing information on Mediterranean children’s preferences and behavior, the present study proposes a co-creation multi-method approach conducted in different stages and in which various professionals interact to improve children’s dietary habits.

## 2. Materials and Methods

### 2.1. Study Protocol

The present study was conducted within the context of the European-funded DELICIOUS project, which study protocol has been recently described [20] and approved by the ethics committee of Mondragon University (no. IEB-20230704).

### 2.2. General Procedure and Methodology

The co-creation process proposed in the present study consisted of a multi-method approach in which five different stages were planned with different points of interaction with children and/or their caregivers.

**Qualitative Data Collection**: This phase involved conducting study trips to the different countries (Portugal, Spain, Italy, Egypt, and Lebanon), in which researchers explored the local environments, market dynamics, and visited different schools. The study trips included conducting interviews with caregivers (teachers, canteen staff and cooks, and parents) to have a general idea on children’s needs, preferences, and challenges in food-related childcare. During the interviews, researchers collected qualitative information on the typical snacks consumed by children as well as their snacking habits. Interviews were conducted by two researchers; notes were taken and later discussed to extract some highlights from each country. The main objectives of these trips and interviews were (1) to identify foods that were less accepted by children, as well as which ones they liked the most (considering the traditional local cuisine); (2) to determine the meal times/number of meals at home vs. school, as well as snacking habits; and (3) to gain a general idea of children’s behavior and participation in tasks such as buying food, cooking, tidying up, and cleaning the kitchen/dining room, etc. Complementing the qualitative data collection, researchers conducted field observations at local markets and grocery stores. These observations aimed to investigate the variety of children’s snacks available and identify potential ingredients for new snack formulations.

**Prototype Design and Development**: Based on the comprehensive insights gathered during the data collection phase, initial product prototypes were developed by the research team (composed of chefs and food scientists). These prototypes were mainly designed to explore children’s perception during the next co-creation phase. The aim of this stage was to create tangible versions of food products that could be made in real-world scenarios, using a variety of ingredients that were available in the different countries (nuts, eggs, vegetables, cereals, seeds, cheese, and chocolate) and resulting in a wide range of flavors and textures (salty, sweet, crunchy, soft, etc.), thus allowing children to interact with different kinds of stimuli and making processes. Six different product prototypes were formulated to be later used in the hands-on activity (Table 1).

**Feedback on Prototypes: Hands-on Activity**. Researchers directly engaged with children through co-creation/cooking sessions, exploring their opinions, reactions, attitudes, and user experiences during the snack-making process. Two workshops were conducted in each country, with 9–12 children/adolescents from 9–14 years old participating in each of them (total n > 100). During the sessions, the team, composed of a chef and a researcher, (1) introduced the aim of the workshop, (2) discussed with the children the different aspects of the Mediterranean diet, (3) talked about their general preferences and food-related habits, and (4) gave the children guidance in cooking the six different snacks after presenting the different ingredients and procedures needed to make each kind of product. Once all snacks were ready, (5) children participated in a tasting activity in which the products were assessed using emojis (each participant had a set of 11 emojis to be used as a check-all-that-apply judgment) [22]. Finally, to determine children’s opinions about the co-creation/cooking workshop, (6) the whole activity was also assessed using the emoji set. Each session lasted approximately 120 min.

**Prototype Optimization**: Considering results from the previous phase, one of the prototypes that was identified as suitable for improvement due to being one of the less-liked snacks was selected (granola bars) and refined, and different flavor options were proposed. The children’s opinions and reactions collected in the previous phase guided improvements, with an emphasis on enhancing the product’s appeal and functionality. The prototype was scaled to industrial level and, therefore, some corrections were made to allow the industrialization process. Table 2 shows the general recipe of the granola bars that were formulated and selected for the last stage of product development, i.e., the cross-cultural assessment. Five different recipes with different nuts and dried fruits were developed, considering the product base, with the aim of testing several flavor combinations: (1) orange and almond (OA), (2) peach and walnut (PW), (3) raisin and walnut (RW), (4) sesame seed and date (SD), and (5) apple with walnut (AW). These nuts and seeds were selected because of their proven nutritional quality [23]; however, the final combinations of nuts, seeds, and dried fruits were determined by the culinary team after testing different combinations to enhance the distinctive flavor profile of each sample.

**Final Product Assessment: Cross-cultural Study**. This stage involved analyzing the five distinct versions of the product, each of them incorporating distinctive ingredients. A comparative study was conducted to determine acceptance using a 7-emoji scale appropriate for children and for the cross-cultural context [24]; in addition, a check-all-that-apply question, including different terms collected during the hands-on activity (ingredients, emotions, consumption contexts), was incorporated into the questionnaire to assess how children perceived each snack version. Over 450 children participated in the tasting study. After removing incomplete questionnaires, a total of 425 responses from the different countries were gathered (participation rate: 23.5% from Egypt, 11.1% from Italy, 17.9% from Lebanon, 30.4% from Portugal, and 17.2% from Spain. Some 54% belonged to the 6–12-year-old group, and 46% belonged to the 13–17-year-old group).

To decide on the specific tasting procedure, the researchers responsible for conducting the snack tasting in each country discussed two possibilities: (a) a real-world scenario in which each child received a bar during a school day, tasted it whenever they were hungry/wanted to eat it, and then answered the corresponding questionnaire (each snack version had to be tested on a different day) or (b) a controlled setting where pieces of the snack samples were monadically served in a classroom and children completed the questionnaire. The second option was selected, and therefore the consumer testing could be considered a centrally located test. Samples were coded and randomized for serving.

**Figure 1:**
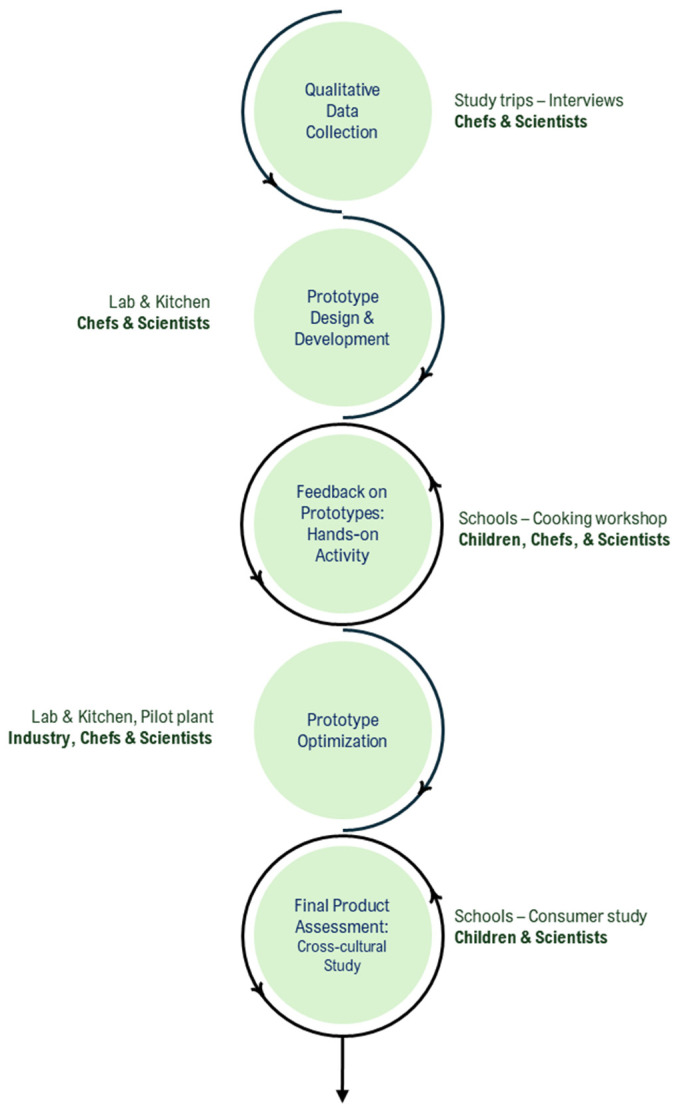
Scheme showing the co-creation multi-method approach with the different stakeholders involved in the process.

Figure 1 summarizes the whole process developed to co-create the snacks, considering the different phases and stakeholders interacting in each of them.

### 2.3. Data Analysis

Qualitative data collected during the study trips, as well as notes on the comments and general behavior of children during the hands-on activity, were gathered by researchers and then discussed in working sessions in order to extract the main information, highlights, and differences among countries. Data about the emoji activity conducted during the hands-on session with children were treated as a check-all-that-apply question. Two contingency tables were built with the data “Country/Emoji” and “Snack/Emoji”, and Fisher’s exact tests were conducted to explore significant differences among countries and snacks. In addition, two correspondence analyses were conducted to explore relationships among variables. Data from the cross-cultural consumer test on the five final granola bars were analyzed by a two-way ANOVA considering sample, country, and their interaction as factors. Differences were considered significant when *p* < 0.05, unless otherwise stated. All statistical analyses were conducted using XLStat (Version, 2021.5, Addinsoft, Denver, CO, USA).

## 3. Results and Discussion

### 3.1. Qualitative Research Responses

The interviews conducted with children’s caregivers (parents and school canteen staff) during phase one provided some general ideas which suggested notable differences in snack preferences and snacking habits across countries.

- Snacks chosen by children tended to be from the sweet category, reflecting a cultural preference for sugary options in Portugal.
- In Spain, common snacks included commercial juices and processed sweets, indicating a trend toward convenience-based choices (e.g., chocolate bars, processed pastries, and ready-to-drink yoghurts).
- Italian children often had access to commercial sweets, packaged sandwiches, and chips, typically available in school kiosks or bars.
- Fresh fruits seemed to be consumed as the primary snack in Egypt, occasionally supplemented by dried fruits such as dates, meaning more natural and minimally processed snacking habits.
- Lebanese children also seemed to favor fresh products, with snacks often including fruits, contributing to a diet with fewer processed snack items.

In addition, snacking moments also varied across countries, influenced by their unique mealtime patterns; because the definition of a snack is “foods that are eaten in between meals”, these differences were foreseeable. These findings from the qualitative phase confirmed the ones obtained during the quantitative study conducted to determine dietary habits in Mediterranean children, in which fruits and commercial biscuits and pastries were the snacks mainly reported by these children’s parents [21].

These data were used to formulate the snack prototypes shown in the Materials and Methods section (Section 2) of the present study during phase two of the co-creation process.

**Figure 2:**
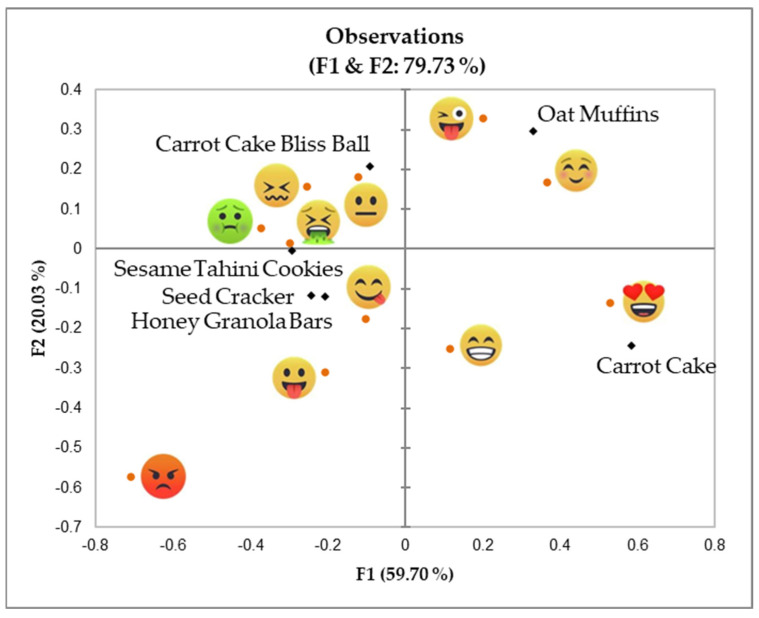
Correspondence analysis results showing the emotional responses across different countries (co-creation/cooking workshop). Legend: orange dots for the current position of emojis; black rhombuses for the current position of snacks.

**Figure 3:**
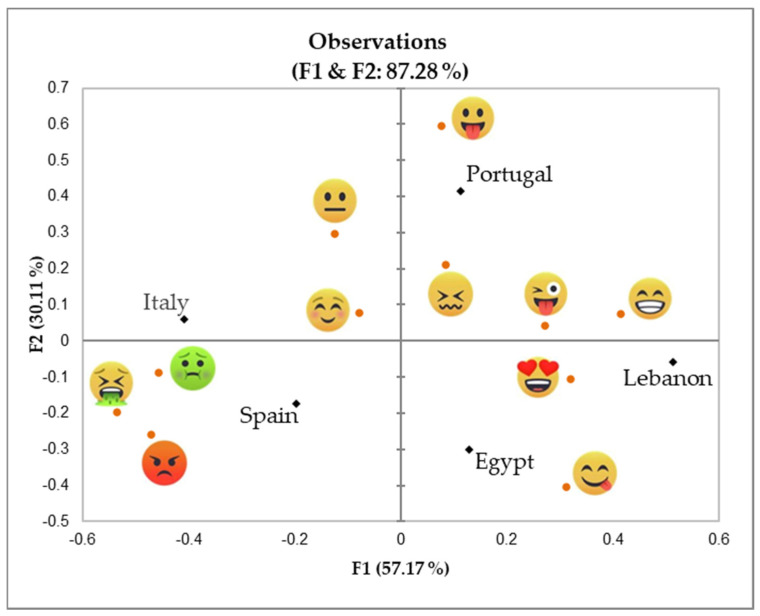
Correspondence analysis results showing the emotional responses across different snacks (hands-on activity). Legend: orange dots for the current position of emojis; black rhombuses for the current position of snacks.

The results of the tasting activity, conducted after making the snacks during the hands-on activity (phase three), are included in Figure 2 and Figure 3, as well as in Table 3, which displays the correspondence analysis with the emotional responses across different snacks and countries and the emojis that were used with higher frequency to assess the snacks in each country, respectively.

Most data variability on the emotional responses elicited by snacks was explained by the first factor (F1), driven by the emojis 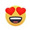 and 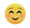, on the right side, and 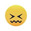, and 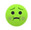 on the left side. Oat muffins and carrot cake were the snacks eliciting more positive emotions, while tahini cookies, seed crackers, and honey granola bars seemed to be the products generating less positive emotions (Figure 1, Table 3). One of the activities/questions included in the moderator’s guide of the hands-on activity was to explore children’s reactions to the different ingredients of the snacks, which were pre-weighed and displayed on the kitchen table. In general, children seemed to be familiar and know all ingredients apart from tahini, which was unfamiliar to some children from Spain, Portugal, and Italy, and some of the seeds from the seed cracker. This lack of familiarity could have influenced their perception of the final snack made with these specific ingredients (sesame tahini cookies and seed crackers). In general, consumer familiarity with a product reduces uncertainty, aligning expectations with reality and increasing acceptance [25,26]. When asked about potential enhancements to these less-liked snacks, children proposed a range of ingredients and sensory modifications; however, no unifying response was obvious, and only “chocolate” seemed to be mentioned by several children as a “flavor-enhancing” solution. This response was not surprising because chocolate has been ranked among children’s favorite foods in Western cultures and in different societies [27].

Interaction with children during the hands-on activity also provided interesting data with which to identify differences in food-related attitudes in each country. Although all children seemed to be happy to spend their time “cooking snacks”, participants from Portugal, Lebanon, and Egypt had a more positive and neophilic attitude to testing new foods, while children in Italy and Spain were more reluctant to try new recipes and ingredients. These impressions were supported by the emojis selected by children to evaluate the snacks (Figure 2), with Portugal, Lebanon, and Egypt predominantly using positive or excited emojis, reflecting their openness to trying the snacks, and Italy and Spain often selecting neutral or negative emojis, indicating a higher level of reluctance to the snacks. Previous studies have shown that emojis are properly used by children to discriminate foods according to valence (positive vs. negative), the power dimension (control vs. lack of control of the situation), and the arousal dimension (high vs. low activation); these results showed that emojis such as 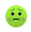 and 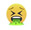 were linked with the emotion “disgust”, 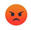 was linked with “angry”, 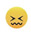 was linked with emotions such as “unhappy” or “disappointed”, 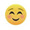 was linked with “calm” and “serene”, and emojis such as 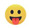, 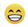, and 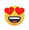 were linked with positive emotions such as “happy” or “in love” [28]. These findings also aligned with the results of previous studies conducted on Italian and Lebanese children [29] and [30], respectively. Di Nucci et al. [29] reported a prevalence of intermediate to high levels of food neophobia among Italian children (42.2 ± 14.0), whereas El Mouallem et al. [30] found lower neophobia scores (39.1 ± 8.3) among children in Lebanon, indicating a low to intermediate neophobia profile. Levels of neophobia have been reported to be critical in children’s adherence to a healthy diet [31] and their acceptance of new snacks [32,33] such as the ones developed for the present study.

In terms of the cooking involvement, some children reported being proactive in assisting their parents with cooking or grocery shopping during the activity, although this was not a general response. The limited participation of children in food- and cooking-related activities could be attributed to societal shifts toward busier lifestyles, which have been linked to a general decline in culinary skills among the population [34,35], thereby limiting children’s opportunities to develop culinary skills at home.

Engaging children in selecting recipes, purchasing ingredients, and preparing meals has been demonstrated to enhance their willingness to taste novel foods and to positively influence their dietary behaviors [36,37,38]. By creating familiarity and positive associations, these activities could reduce their hesitance toward less familiar food options. During the hands-on activity conducted in the present research (phase three), all participants were active in processing the different ingredients and making the new snacks, although a small percentage of them did not want to test the final products. Before cooking, the children’s initial willingness to try the snacks was notably lower than their final willingness to try them after participating. Cooking and having family meals have been suggested to significantly impact children’s and adolescents’ acquisition of relational food literacy competencies [39]. The results of this intervention reinforced the idea that involving children in hands-on cooking activities increased their willingness to try new products, although higher participation could be needed to confirm this result, and a follow-up control would be needed to determine if children’s engagement with food and cooking lasts with time.

### 3.2. Cross-Cultural Study: Quantitative Responses

One of the snacks identified as less liked during the hands-on activity (granola bars) was later optimized, and five versions were designed using ingredients from the different countries as inspiration; these were scaled to the pilot plant. The results of the two-way ANOVA from the cross-cultural evaluation of the five distinct granola bar versions revealed significant differences in the 7-emoji appreciation scores across countries and granola bars (*p*-value < 0.0001). Portuguese, Spanish, Lebanese, and Egyptian children reported similar liking scores for the snacks tested, while Italian children showed significantly lower liking scores for all samples. This finding supports the hypothesis generated during the qualitative phase, which suggested that Italian children were generally more neophobic than their counterparts from other countries. This neophobic tendency was observed during the hands-on activity, where Italian participants were less willing to try the developed snacks (novel recipes or unfamiliar ingredients).

Regarding the granola bars, PW received significantly higher liking scores than RW and AW. These results indicated a clear preference for some of the tested samples, with PW being the most liked and AW being the least liked granola bar across all countries. Table 4 shows the results of the one-way ANOVA between granola bars by country, showing cross-cultural differences in sample preferences (the two-way ANOVA results on the interaction “country × sample” showed significant differences, *p*-value < 0.0001). PW was one of the most liked granola bars, particularly in Spain. RW stood out as the most favored in Portugal, while SD was the top-rated granola bar in Lebanon, and AW was the most liked in Egypt. No significant differences were observed among the granola bars in Italy.

**Figure 4:**
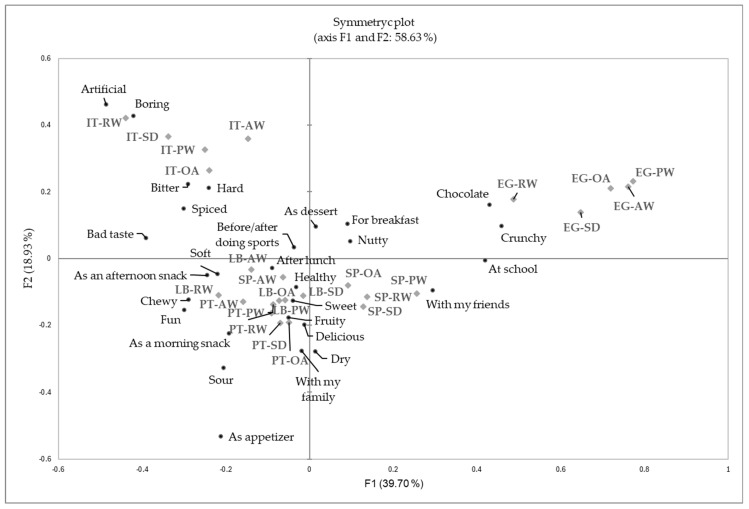
Symmetric plot (correspondence analyses) of the CATA responses showing differences among granola bars in the different studied countries (cross-cultural study).

Figure 4 presents a symmetric plot resulting from a correspondence analysis (CA) of the CATA (check-all-that-apply) responses, illustrating the differences in how granola bars were perceived across the studied countries. The two dimensions (F1 and F2) accounted for 58.6% of the variability in the data, with F1 explaining 39.7% and F2 explaining 18.9%. Distinct clustering of granola bars associated with specific attributes by country was observed. The results of the correspondence analysis reinforced the idea that children’s food preferences were influenced by personal predispositions as well as the food environment and culture.

Positive terms such as “Chocolate” and “Crunchy” were strongly associated with granola bars from Egypt (EG), reflecting children’s natural preference for sweet and texturally appealing foods, as noted by Birch [40]. Contextual mentions such as “At school” and “With my friends” were linked to the perception of granola bars by both Egyptian (EG) and Spanish (SP) children, emphasizing their appeal in these social settings. This highlights the significant role of peers in shaping children’s food choices, particularly in group settings where social influences are highly impactful [41]. Strong positive correlations were observed between attributes such as “Crunchy” and “Nutty”, “Sweet” and “Delicious”, as well as “At school” and “With my friends”, suggesting that these combinations represented favorable sensory and situational perceptions. Attributes such as “Soft”, “Fun”, and “As a morning/afternoon snack” were more frequently associated with granola bars by children from Portugal (PT) and Lebanon (LB), suggesting their suitability for relaxed or familiar contexts. Sick et al. [42] suggested that emotions elicited by foods in specific contexts could play a crucial role in food acceptance and choice. Therefore, considering the context “As a morning/afternoon snack” could improve the emotional salience of certain granola bars, making them more enjoyable for children.

On the other hand, negative attributes such as “Artificial”, “Boring”, and “Bad taste” clustered around granola bars tested by children from Italy (IT), highlighting the less favorable perceptions among these young consumers. This finding aligns with previous evidence of higher levels of neophobia among Italian children, likely due to limited exposure or lack of familiarity with such products [40].

Some of the main limitations of the present study are linked to the lack of characterization on the current background of children participating in the research. Although all settings were similar and specific common guidelines were used to deploy the activities in the different countries, no questionnaires were used to determine the baseline food- and cooking-related knowledge of children. A pre-selection phase to standardize the population participating in the co-design workshops could be used to improve the proposed methodology and better understand cross-cultural differences. In addition, children’s familiarity with the ingredients used in the hands-on activity may have influenced their responses. Although most ingredients can be found in all the countries in which the study was developed, some of them and their flavors are more common in specific countries (e.g., tahini used in the sesame tahini cookies recipe or mascarpone cheese used in the oat muffins recipe). Further studies should include specific questionnaires to address this limitation and consider previous exposure or familiarity with the ingredients.

## 4. Conclusions

Co-creation activities with children proved to be effective and insightful in developing healthier snack options that addressed regional preferences, and the present study shows a multi-method approach to designing new foods considering children’s opinions and needs. The findings presented in this manuscript will be valuable for researchers engaged in food design research and those investigating strategies through which to improve children’s dietary habits, particularly in regions with an emphasis on Mediterranean diet adherence. By involving children directly in the creation and evaluation process, different product prototypes were designed to be appealing and to increase consumption of specific ingredients typical of the Mediterranean diet (e.g., nuts, seeds, cereals). Children’s snack preferences and general food-related attitudes were found to be influenced by a combination of innate sensory predispositions, cultural norms, and contextual factors. Among these, neophobia and familiarity with the product emerged as critical considerations in children’s product development. Children from Portugal, Lebanon, and Egypt demonstrated a more positive and neophilic attitude towards new foods compared to children from Italy and Spain, which resulted in a less favorable perception and lower acceptance of all the developed prototypes by Italian children. Emojis used by children reflected these differences, with positive emojis being more prevalent in Portugal, Lebanon, and Egypt and neutral/negative emojis more common in Italy and Spain. The cross-cultural evaluation of granola bars showed that the peach and walnut (PW) flavor was the most preferred across countries, highlighting its potential as a healthier alternative to low-nutrition commercial snacks. This study underscores the importance of adapting product development to regional tastes and cultural preferences to maximize acceptance as well as the value of co-creation in addressing dietary challenges and promoting healthier eating habits among children. Involving children in hands-on cooking activities, such as preparing snacks, can significantly increase their willingness to try new and unfamiliar foods and increase their food literacy levels. Future research should explore the long-term impact of these interventions on dietary adherence, eating behaviors, and health outcomes.

## Figures and Tables

**Table 1:** Ingredient composition of the different prototypes used during the hands-on activity with children. Ingredients were bought in grocery stores in each country, ensuring availability.

Oat Muffins		Sesame Tahini Cookies		Carrot Cake Bliss Balls	
%	Ingredient	%	Ingredient	%	Ingredient
41%	Mascarpone cheese	35%	Almond flour	29%	Sunflower seeds
20%	Eggs	26%	Honey	29%	Shredded coconut
12%	Oatmeal flakes	18%	Sesame seeds	25.4%	Dates
9.8%	Cashews	18%	Tahini	13%	Carrots
9.8%	Dark chocolate chunks	1%	Vanilla extract	2%	Maple syrup
6%	Cane sugar	0.7%	Baking soda	1%	Salt
1.3%	Baking powder	1%	Sea salt	0.6%	Cinnamon
0.5%	Salt				
**Carrot Cake**		**Seed Crackers**		**Honey Granola Bar**	
**%**	**Ingredient**	**%**	**Ingredient**	**%**	**Ingredient**
25.6%	Carrot	56.5%	Water	26.4%	Oatmeal flakes
22%	All-purpose flour	12.4%	Pumpkin seeds	23.3%	Hazelnuts
15%	Eggs	12.4%	Sesame seeds	13.2%	Dried fruit mix
14.5%	Brown sugar	5.6%	Chia seeds	13%	Honey
12.8%	Vegetable oil	4.5%	Poppy seeds	9%	Butter
9.2%	Hazelnuts	4.5%	Flax seeds	6.3%	Dark brown sugar
0.2%	Salt	2.3%	Oatmeal	6.3%	Sunflower seeds
0.2%	Cinnamon and nutmeg	1%	Salt	1%	Salt
0.2%	Vanilla extract	0.4%	Cumin	1%	Vanilla extract
0.15%	Baking powder	0.4%	Garlic powder	0.5%	Cinnamon
0.15%	Baking soda				

**Table 2:** Ingredient composition of the different prototypes used during the hands-on activity with children. Legend: nut and seed percentages varied by sample to sum the total 100%.

%	Ingredient	Detail on Ingredients
14–19%	Nuts	Hazelnuts, walnuts, almond
18%	Cereals	Oat, sorghum, quinoa, rice
12–17%	Seeds	Pumpkin, sunflower, sesame
16%	Honey	
11%	Butter (unsalted)	
9%	Dried fruit	Peach, date, orange, apple, raisin
7%	Brown sugar	
5%	Chocolate cover	>70% cocoa
2%	Olive pulp extract	
1%	Salt and spices	Cinnamon, vanilla extract

**Table 3:** Fisher’s exact test results showing the emojis used with higher frequency (*p*-value < 0.05) for each snack and country. Samples that did not exhibit significant differences in emoji frequency were omitted from the table.

Country	Snack	Emoji
Lebanon	Carrot Cake	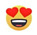
	Oat Muffins	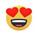
	Sesame Tahini Cookies	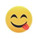
	Seed Crackers	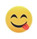
Portugal	Carrot Cake Bliss Balls	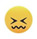
	Carrot Cake	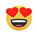 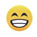
	Honey Granola Bars	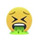
Egypt	Carrot Cake Bliss Balls	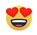 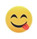
	Carrot Cake	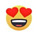 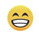
	Honey Granola Bars	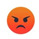
	Oat Muffins	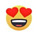
	Sesame Tahini Cookies	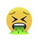
Spain	Seed Crackers	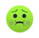
Italy	Carrot Cake Bliss Balls	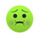 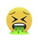
	Carrot Cake	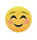
	Oat Muffins	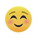
	Sesame Tahini Cookies	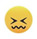
	Seed Crackers	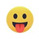

**Table 4:** Two-way ANOVA results of the 7-emoji appreciation score (means) of granola bars across countries and snack types. Different letters indicate significant differences among interactions (Tukey’s HSD).

Snack	Spain	Portugal	Lebanon	Egypt	Italy
PW	5.1 a	4.3 a	4.6 a	4.1 ab	3.2
SD	4.5 a	4.4 a	4.7 a	4.1 ab	2.8
OA	4.5 a	4.3 a	4.3 ab	4.2 ab	3.0
RW	4.4 a	4.8 a	4.1 ab	3.7 b	2.5
AW	3.0 b	3.7 b	3.7 b	4.3 a	2.6
*p*-value	<0.0001	<0.0001	0.005	0.024	0.202

## Data Availability

The original contributions presented in this study are included in the article. Further inquiries can be directed to the corresponding author.
